# Job burnout among public health practitioners in urban China: insights from the post-COVID-19 pandemic context

**DOI:** 10.3389/fpubh.2025.1518114

**Published:** 2025-05-21

**Authors:** Ping Xu, Qing Fang, Shasha Yuan, Na Zhang, Danlei Wang, Zhongyue Huang, Min Xian

**Affiliations:** ^1^Public Health Policy Research Office, Baoan District Center for Disease Control and Prevention, Shenzhen, Guangdong, China; ^2^Public Health Strategic Intelligence Research Office, Institute of Medical Information, Chinese Academy of Medical Sciences and Peking Union Medical College, Beijing, China

**Keywords:** job burnout, public health practitioners, China, post-COVID-19, influencing factors

## Abstract

**Background:**

Job burnout is particularly prevalent within the healthcare sector, with public health practitioners (PHPs) being especially vulnerable. The global impact of the coronavirus disease 2019 (COVID-19) pandemic has been profound, yet the prevalent level of job burnout among PHPs following the crisis has been largely overlooked. This study aims to assess the prevalence and determinants of job burnout among PHPs in the post-COVID-19 era, thereby providing a theoretical foundation for the development of targeted interventions.

**Methods:**

This cross-sectional survey was conducted from July to October 2023, targeting members of the Center for Disease Control and Prevention and the Public Health Service Center in Baoan District, Shenzhen. A non-random convenience sampling was employed to recruit 222 participants. Demographic and work-related information was compiled. Job burnout was assessed with Chinese revised version of the Maslach Burnout Inventory-General Survey. Binary logistic regression analysis was employed to identify factors influencing job burnout among participants. The mediation effect was tested using the bias-corrected percentile Bootstrap method with 5,000 resamples.

**Results:**

The prevalence of job burnout among the PHPs was found to be 50.90%, with rates of mild, moderate, and severe burnout at 27.03, 15.32, and 8.56%, respectively. Multivariable analysis indicated that self-rated mental health (*OR* = 0.436, 95% *CI*: 0.230, 0.827), workload intensity (*OR* = 5.183, 95% *CI*: 1.751, 15.340), and the family support for work (*OR* = 3.313, 95% *CI*: 1.335, 8.222) were significantly associated with burnout (*p* < 0.05). The PHPs exhibiting poorer self-rated mental health, higher workload, and lower family support for work were at greater risk of job burnout. The mediation analysis revealed that elevated workload indirectly increased the likelihood of burnout (indirect effect = 2.931, 95% *CI*: 1.111, 4.750), exhaustion dimension (indirect effect = 2.801, 95% *CI*: 1.115, 4.486) and cynicism dimension (indirect effect = 2.977, 95% *CI*: 1.127, 4.826) by exacerbating mental health deterioration.

**Conclusion:**

Job burnout has emerged as a common concern among the PHPs in the aftermath of the COVID-19 pandemic. To effectively address burnout, it is crucial to develop effective intervention measures aimed at mitigating risk factors, ultimately enhancing the well-being of the PHPs.

## 1 Introduction

The phenomenon of burnout has a longstanding history. The term “burnout” was first introduced in a novel by the author Graham Greene. Subsequently, Herbert Freudenberger popularized the concept within the psychological domain, focusing on its identification, treatment, and prevention rather than formal assessment (1, 2). Through extensive empirical research, Maslach and collaborators reconceptualized burnout as a psychological syndrome caused by the prolonged response to chronic interpersonal stress sources within occupational contexts. It is characterized by three core dimensions: overwhelming exhaustion, feelings of cynicism and detachment from the job, and a sense of ineffectiveness and lack of accomplishment (3). The exhaustion dimension encompasses profound fatigue, depletion of emotional resources, and psychophysical weariness. Cynicism originally termed depersonalization, and this dimension manifests as hostility, withdrawal, loss of idealism, or dysfunctional interpersonal responses to service recipients. The inefficacy dimension is characterized by decreased productivity, impaired coping abilities, low morale, and an eroded sense of achievement (3). Moreover, Maslach and collaborators developed the Maslach Burnout Inventory, a self-report questionnaire widely utilized to assess burnout (2). Although alternative definitions and conceptualizations of burnout have been proposed by other researchers, the model advanced by Maslach and Jackson remains the most extensively validated and accepted across a wide range of countries and professions (4).

Job burnout exists in various industries (5, 6), with the medical domain demonstrating a noteworthy incidence. Investigations have underscored a heightened vulnerability to job burnout among public health practitioners (PHPs) compared to their clinical counterparts (7). This disproportionality may be attributed to factors such as the comparatively marginalized societal status, diminished sense of professional attainment, and substantial remuneration gap between the PHPs and clinical physicians in China. Serving as “gatekeepers” of public health, these professionals grapple with substantial occupational demands pertaining to emergency preparedness, infectious disease monitoring, epidemic surveillance, and chronic ailment management, etc. The outbreak of the coronavirus disease 2019 (COVID-19) pandemic intensified their workload, propelling them into high-stress environments where their mental well-being was significantly challenged, and predisposing them to job burnout.

Research has shown that job burnout among the PHPs is common. A cross-study indicated the incidence of job burnout of three-tiered PHPs in China was 67.55% (8). In a meta-analysis, the investigators found prevalence of job burnout ranged from 2.5 to 87.9% in different countries (9). The ramifications of job burnout extend beyond personal well-being, moreover, it has been associated with work efficiency, job satisfaction, and even staff turnover among the PHPs (10–12). The professional status of these individuals not only impacts their personal mental and physical health but critically intersects with the efficacy of public health interventions and the broader health outcomes of the population they serve. The previous studies further highlighted that burnout among PHPs was influenced by multifaceted determinants. These includes factors intrinsic to the profession itself, such as prolonged working hours, excessive workload, and elevated job stress (7, 13, 14), as well as individual-level characteristics (e.g., educational attainment, professional title, income) (15, 16), challenges in managing interpersonal dynamics, organizational policy frameworks (17), and external contextual elements (e.g., familial support) (18).

While the global landscape has been profoundly altered by the COVID-19 pandemic (19), scholarly inquiries have predominantly focused on job burnout dynamics during the acute phases of the crisis rather than post-pandemic periods (20–24). With the World Health Organization’s proclamation on May 5, 2023, that COVID-19 no longer constituted a public health emergency of international concern (25), inquiries into the post-pandemic job burnout among the PHPs remain scarce. Consequently, this study centers attention on the PHPs within a district of Shenzhen, a city located in southern China to clarify the job burnout situation in the post-COVID-19 era, identify its key determinants, and further delineate the processes and mechanisms by which these factors contribute to burnout through mediating effects, thereby providing pertinent insights to enhance public health practices, fortifying the occupational well-being of PHPs and the foundations of the public health framework.

## 2 Methods

### 2.1 Study participants

This cross-sectional study was conducted from July to October 2023 in Baoan District, Shenzhen. Public health institutions within the district include the Disease Prevention and Control Center, Public Health Service Center and subordinate street-level branches in Baoan District, Shenzhen. PHPs in this study are individuals who are professionally engaged in disease prevention, health promotion, epidemiological surveillance, the formulation and implementation of health policies, environmental health management, public health emergency response, and related activities within above-mentioned public health institutions. The participants primarily comprised establishment-based personnel—individuals holding government-sanctioned positions secured through competitive public recruitment examinations, with long-term statutory contracts ensuring job security and public-sector welfare benefits—and post-quota system employees, an intermediate employment category where total authorized positions and remuneration budgets are centrally regulated by superior authorities, but managed locally, offering reduced tenure stability and welfare entitlements compared to establishment-based personnel. Within the institutions, they engaged in health technical positions, including public health physicians, laboratory technicians, and health education specialists responsible for specialized tasks such as disease prevention and health promotion, or non-health technical positions, encompassing administrative staff managing institutional operations and logistical personnel supporting technical workflows.

The PHPs from these institutions were selected to participate in an online survey. Inclusion criteria were: (1) current employees of public health institutions; (2) more than 1 year of work experience in public health; and (3) informed consent for participation in this study. Exclusion criteria included: having ceased regular duties (e.g., on study leave or secondment to other institutions) for 6 months or more.

### 2.2 Questionnaire development

Prior to formal survey administration, semi-structured interviews were conducted with a purposive sample of professionals from participating research institutions to gain preliminary qualitative insights into the prevalence and multi-level determinants of burnout among the PHPs. Building upon these emergent themes and aligned with theoretical frameworks identified in systematic literature reviews, a draft questionnaire was iteratively developed. This preliminary instrument underwent two rounds of expert consultation. A pre-survey was conducted at the Baoan District Center for Disease Control to further tailor the questionnaire based on feedback and suggestions from respondents, yielding the final version. The survey included: (1) basic sociodemographic characteristics such as gender, age, marital status, education level, professional title, years of work, and position; (2) potential factors influencing job burnout, involving individual work context (such as work intensity), interpersonal dynamics (such as the relationship with colleagues and superiors), organizational policy frameworks (such as professional title promotion mechanism), and external contextual elements (such as family support); and (3) the Chinese revised version of the Maslach Burnout Inventory-General Survey (MBI-GS) (26).

### 2.3 Data collection

From July to October 2023, non-random convenience sampling was employed to recruit survey participants from public health institutions. The anonymous questionnaire was sent via an online platform Survey Star (Changsha Ran Xing Science and Technology, Shanghai, China) to the participants.

### 2.4 Measurement and determination of job burnout

Since the study participants did not engage in direct patient contact, the Maslach Burnout Inventory–Human Services Survey, typically applied in healthcare contexts (27), was not prioritized. The Chinese revised version of MBI-GS, a 15-item scale revised by Li and colleagues, has been validated for high reliability and validity in burnout studies across multiple industries in China (28). Therefore, the measurement of job burnout was conducted using the Chinese revised version of MBI-GS. Each item in this scale was rated on a Likert scale from 0 to 6, ranging from “never” to “every day.” The scale encompasses three dimensions: exhaustion, cynicism and inefficacy. For each dimension, scores are calculated as the total sum of the individual item scores. The first two dimensions, exhaustion and cynicism, apply a positive scoring methodology, whereby higher scores correspond to elevated levels of job burnout. In contrast, the inefficacy dimension utilizes a reverse scoring approach, wherein lower scores signify greater levels of job burnout. This nuanced scoring system facilitates a comprehensive assessment of the multifaceted nature of job burnout in the workplace. The cut-off values for job burnout were determined by the upper third percentile of scores for each dimension (23). In this study, the cut-off value of exhaustion dimension was 11 points, cynicism dimension was 8 points, and inefficacy dimension was 17 points. Based on the scores, no burnout was defined as not exceeding the cut-off in any dimension, mild burnout as exceeding the cut-off in one of the dimensions, moderate burnout as exceeding in two, and severe burnout as exceeding in all three dimensions. Individuals classified with mild, moderate, or severe burnout were considered to be burnout (23). The scale exhibited robust internal consistency across its dimensions. Specifically, Cronbach’s *α* coefficients for the subscales of exhaustion, cynicism and inefficacy were 0.951, 0.931, and 0.917, respectively. The overall scale demonstrated excellent reliability with a Cronbach’s *α* coefficient of 0.855, confirming strong internal consistency both globally and at the subscale level.

### 2.5 Data analysis

In the descriptive section, frequency and proportions were used. Chi-square tests were used to compare the job burnout of the PHPs with different characteristics. A forward stepwise model selection approach, based on standard likelihood ratio was applied to the binary logistic regression analysis to identify the factors influencing job burnout among respondents. The significance of mediation effect was tested using the bias-corrected percentile Bootstrap method with 5,000 resamples. A *p*-value < 0.05 was considered statistically significant; and all tests were two-tailed. Statistical analyses were conducted using two software platforms: SPSS (version 26) was employed for descriptive statistics, Chi-square tests, and logistic regression analysis, while Stata 16 was utilized for mediation effect analysis.

## 3 Results

### 3.1 Demographic and work-related characteristics of the participants

A total of 228 individuals were surveyed. Due to logical errors in the questionnaire or other reasons, 6 unqualified questionnaires were excluded, and the qualification rate of the questionnaire was 97.37%. Ultimately, 222 individuals were included in the analysis. The demographic characteristics of the participants were presented in Table 1. The sample comprised 126 females (56.76%). The age range spanned from 22 to 62 years old, with the largest proportion (36.49%) belonging to age group of 20–29 years old. The majority of participants held a bachelor’s degree (72.97%), followed by a master’s degree (13.06%). In terms of marital status, 61.71% were married. Regarding the discipline area, public health was most common (56.31%).

**Table 1:** Demographic characteristics of participants.

Variables	Categories	Frequency (*N* = 222)	Percentage (%)
Sex, *n* (%)	Male	96	43.24
	Female	126	56.76
Age, *n* (%)	20–29	81	36.49
	30–39	54	24.32
	40–49	59	26.58
	≥50	28	12.61
Marital status, *n* (%)	Married	137	61.71
	Others	85	38.29
Number of children, *n* (%)	0	94	42.34
	1	67	30.18
	≥2	61	27.48
Academic qualifications, *n* (%)	College degree and below	29	13.06
	Undergraduate degree	162	72.97
	Postgraduate and above	31	13.96
Discipline area^a^, *n* (%)	Public health	125	56.31
	Others	97	43.69
Annual post-tax income, *n* (%)	≤ 150,000 RMB	91	40.99
	> 150,000 RMB	131	59.01
Ideal annual income, *n* (%)	Increase by ≤ 50% compared to current income	155	69.82
	Increase by > 50% compared to current income	67	30.18

a Discipline area refers to the academic major of the individuals. In China, individuals with public health professional backgrounds or other medical backgrounds such as clinical medicine and nursing are able to engage in public health practice.

**Figure 1:**
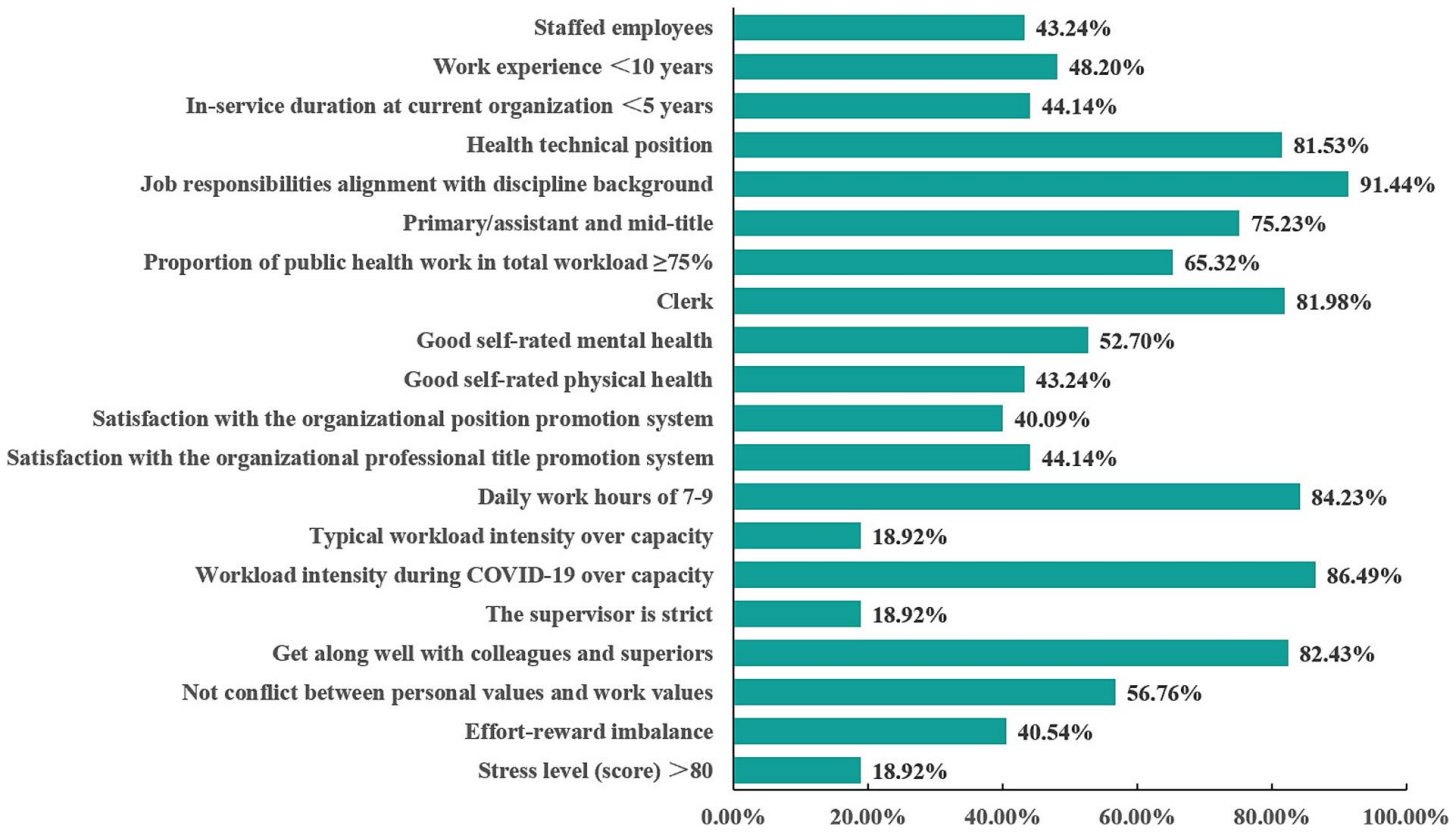
Work-related characteristics of the participants.

As shown in Figure 1, 48.20% of participants were employed for less than 10 years, and 44.14% had served fewer than 5 years at their current organization. A majority (81.53%) held health technical positions. Public health tasks constituted ≥ 75% of the total workload for about two-thirds of participants (65.32%), while daily work hours were standardized to 7–9 h for 84.23%. 18.92% reported workload intensity exceeding their capacity. 40.09% expressed satisfaction with organizational position promotion systems and 44.14% with professional title promotion mechanisms.

### 3.2 Prevalence of burnout among the PHPs

**Figure 2:**
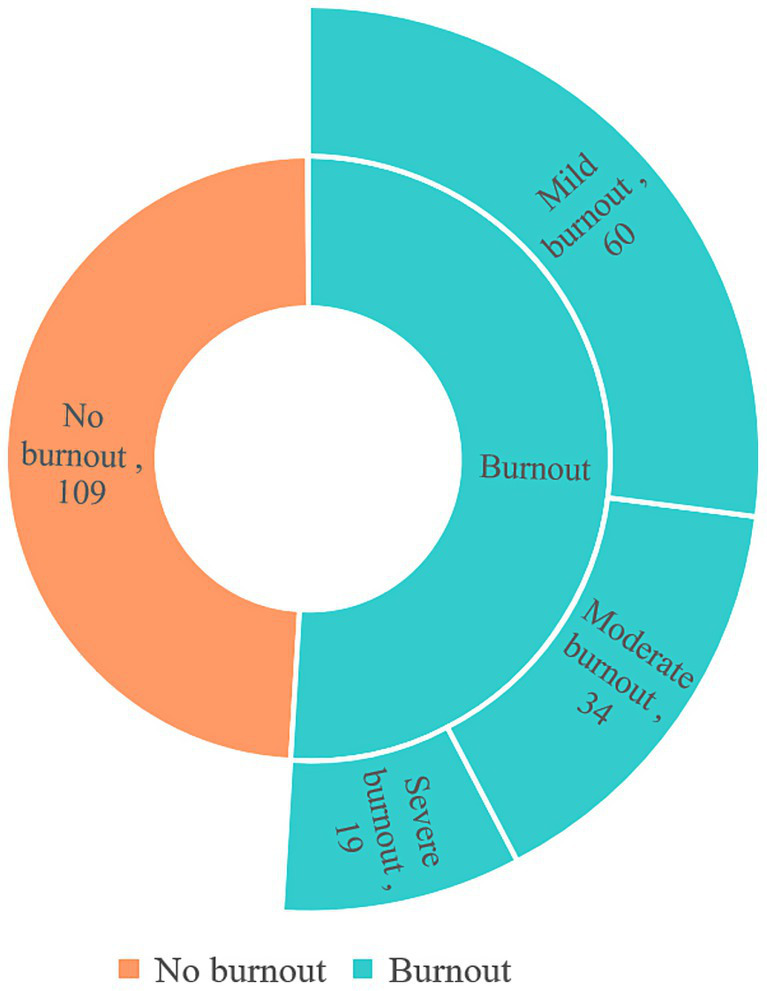
Job Burnout status among the public health practitioners.

The job burnout status was shown in Figure 2. Among the 222 survey participants, 113 were identified with job burnout, with a prevalence of 50.90%. Among them, 60 (27.03%) participants experiencing mild burnout, 34 (15.32%) with moderate burnout, and 19 (8.56%) demonstrating severe burnout. From diverse perspectives, 32.88% of participants reported exhaustion, 21.62% displayed cynicism phenomenon, and 28.83% acknowledged feelings of inefficacy. The prevalence of subcomponents was outlined in Table 2.

**Table 2:** MBI-GS score and prevalence of the subcomponents of burnout among the PHPs.

Subcomponents	Score [M (P25, P75)]	Prevalence (%)
Exhaustion	10.00 (7.00, 14.00)	32.88 (73/222)
Cynicism	6.00 (4.00, 8.00)	21.62 (48/222)
Inefficacy	18.00 (16.00, 24.25)	28.83 (64/222)

M, median; MBI-GS, Maslach Burnout Inventory-General Survey; PHPs, public health practitioners; P25, 25th percentile; P75, 75th percentile.

### 3.3 Single factor analysis of burnout of the PHPs

The Chi-square test was used to compare the prevalence of job burnout among the PHPs with different characteristics (Table 3). The results showed that there were statistically significant differences in the prevalence of job burnout among the PHPs with different levels of professional title, ideal annual income, self-rated mental health, self-rated physical health, satisfaction with the organizational position promotion mechanism, satisfaction with the organizational professional title promotion mechanism, typical workload intensity, the rapport with colleagues and superiors, conflict between personal values and work values, effort-reward imbalance, family support for work, the work-life balance, and stress level (*p* < 0.05).

**Table 3:** Univariate analysis of the associated factors of burnout among the PHPs.

Variables	Categories	Burnout (*n* = 113)	No burnout (*n* = 109)	*χ* ^2^	*p*
Sex	Male	49 (51.04)	47 (48.96)	0.001	0.971
	Female	64 (50.79)	62 (49.21)		
Age	20–29	42 (51.85)	39 (48.15)	3.330	0.343
	30–39	32 (59.26)	22 (40.74)		
	40–49	28 (47.46)	31 (52.54)		
	≥50	11 (39.29)	17 (60.71)		
Marital status	Married	63 (54.01)	74 (45.99)	3.459	0.063
	Others	50 (58.82)	35 (41.18)		
Number of children	0	53 (56.38)	41 (43.62)	2.005	0.367
	1	32 (47.76)	35 (52.24)		
	≥2	28 (45.90)	33 (54.10)		
Academic qualifications	College degree and below	12 (41.38)	17 (58.62)	1.440	0.487
	Undergraduate degree	86 (53.09)	76 (46.91)		
	Postgraduate and above	15 (48.39)	16 (51.61)		
Discipline area^a^	Public health	64 (51.20)	61 (48.80)	0.010	0.919
	Others	49 (50.52)	48 (49.48)		
Professional title, *n* (%)	Senior	9 (36.00)	16 (64.00)	9.859	0.007
	Primary/assistant and mid-title	95 (56.89)	72 (43.11)		
	No title	9 (30.00)	21 (70.00)		
Employment types	Establishment-based personnel	42 (54.55)	35 (45.45)	0.752	0.687
	Post-quota system employees	46 (47.92)	50 (52.08)		
	Others	25 (51.02)	24 (48.98)		
Work experience	<10 years	60 (56.07)	47 (43.93)	2.322	0.313
	10–20 years	29 (47.54)	32 (52.46)		
	>20 years	24 (44.44)	30 (55.56)		
In-service duration at current organization	<5 years	46 (46.94)	52 (53.06)	4.480	0.106
	5–10 years	20 (68.97)	9 (31.03)		
	≥10 years	47 (49.47)	48 (50.53)		
Position types	Health technical position	96 (53.04)	85 (46.46)	1.792	0.181
	Non-health technical position	17 (41.46)	24 (58.54)		
Position titles	Clerk	95 (52.20)	87 (47.80)	0.680	0.410
	Non-clerk^b^	18 (45.00)	22 (55.00)		
Job responsibilities alignment with discipline background	Yes	100 (49.26)	103 (50.74)	2.552	0.110
	No	13 (68.42)	6 (31.58)		
Proportion of public health work in total workload	≥75%	68 (46.90)	79 (53.10)	2.682	0.101
	<75%	45 (58.44)	32 (41.56)		
Annual post-tax income	≤150,000 RMB	53 (58.24)	38 (41.76)	3.325	0.068
	>150,000 RMB	60 (45.80)	71 (54.20)		
Ideal annual income, *n* (%)	Increase by ≤50% compared to current income	69 (44.52)	86 (55.48)	7.552	0.006
	Increase by >50% compared to current income	44 (65.67)	23 (34.33)		
Self-rated mental health, *n* (%)	Poor	17 (85.00)	3 (15.00)	48.159	<0.001
	Fair	62 (72.94)	23 (27.06)		
	Good	34 (29.06)	83 (70.94)		
Self-rated physical health, *n* (%)	Poor	14 (87.50)	2 (12.50)	32.162	< 0.001
	Fair	70 (63.64)	40 (36.36)		
	Good	29 (30.21)	67 (69.79)		
Satisfaction with the organizational position promotion mechanism, *n* (%)	Satisfied	26 (29.21)	63 (70.79)	28.472	< 0.001
	Neutral	67 (63.81)	39 (36.19)		
	Dissatisfied	20 (71.43)	8 (28.57)		
Satisfaction with the organizational professional title promotion mechanism, *n* (%)	Satisfied	32 (32.65)	66 (67.35)	25.848	< 0.001
	Neutral	64 (62.14)	39 (37.86)		
	Dissatisfied	17 (80.95)	4 (19.05)		
Average daily working hours	<7 h	11 (55.00)	9 (45.00)	5.792	0.055
	7–9 h	90 (48.13)	97 (51.87)		
	>9 h	12 (80.00)	3 (20.00)		
Typical workload intensity, *n* (%)	Within capacity	76 (42.22)	104 (57.78)	26.868	< 0.001
	Over capacity	37 (88.10)	5 (11.90)		
Workload intensity during COVID-19	Within capacity	11 (36.67)	19 (63.33)	2.812	0.094
	Over capacity	102 (53.13)	90 (46.88)		
Whether the supervisor is strict or not	Yes	26 (61.90)	16 (38.10)	2.510	0.113
	No	87 (48.33)	93 (51.67)		
Get along well with colleagues and superiors or not, *n* (%)	Yes	85 (46.45)	98 (53.55)	7.281	0.007
	Neutral or not	28 (71.79)	11 (28.21)		
Conflict between personal values and work values, *n* (%)	Not conflict	44 (34.92)	82 (65.08)	29.777	< 0.001
	Neutral	61 (71.76)	24 (28.24)		
	Conflict	8 (72.73)	3 (27.27)		
Effort-reward imbalance, *n* (%)	Yes	57 (63.33)	33 (36.67)	9.448	0.002
	No	56 (42.42)	76 (57.58)		
Family support for work, *n* (%)	Supportive	70 (40.94)	101 (59.06)	29.577	< 0.001
	Average or non-supportive	43 (84.31)	8 (15.69)		
Whether the work-life balance be achieved or not, *n* (%)	Yes	93 (46.27)	108 (53.73)	18.244	< 0.001
	No	20 (95.24)	1 (4.76)		
Stress level (score), *n* (%)	≤60	40 (37.38)	67 (62.62)	21.133	< 0.001
	61–80	40 (54.79)	33 (45.21)		
	>80	33 (78.57)	9 (21.43)		

PHPs, public health practitioners.

a Discipline area refers to the academic major of the individuals. In China, individuals with public health professional backgrounds or other medical backgrounds such as clinical medicine and nursing are able to engage in public health practice.

b Their roles comprise section-level leadership (e.g., department directors) and institutional-level leadership (e.g., organizational executives).

### 3.4 Binary logistic regression analysis on influencing factors of burnout among the PHPs

Taking the job burnout as the dependent variable, all the factors with *p* < 0.20 in the univariate analysis were included in the multivariable logistic regression model (variable assignment is shown in Table 4). The multivariable analysis indicated that self-rated mental health, workload intensity, and family support for work were associated with the job burnout. A better self-rated mental health correlated with a lower probability of job burnout (*OR* = 0.436, 95% *CI*: 0.230, 0.827). The PHPs experiencing work overload were 5.183 times more likely to encounter job burnout compared to their counterparts without overload (*OR* = 5.183, 95% *CI*: 1.751, 15.340). Furthermore, lower levels of family support for work were associated with a higher likelihood of job burnout (*OR* = 3.313, 95% *CI*: 1.335, 8.222). The factors associated with job burnout were presented in Table 5.

**Table 4:** Variables configuration and assignment.

Variables	Assignment of variables
Marital status	1 = Married, 2 = Others
Professional title	1 = Senior, 2 = Primary/assistant and mid-title, 3 = No title
In-service duration at current organization	1 = <5 years, 2 = 5–10 years, 3 = ≥ 10 years
Position types	1 = Health technical position, 2 = Non-health technical position
Job responsibilities alignment with discipline background	1 = Yes, 2 = No
Proportion of public health work in total workload	1 = ≥ 75%, 2 = < 75%
Annual post-tax income	1 = ≤ 150,000 RMB, 2 = > 150,000 RMB
Ideal annual income	1 = increase by ≤ 50% compared to current proportion, 2 = increase by >50% compared to current proportion
Self-rated mental health	1 = Poor, 2 = Fair, 3 = Good
Self-rated physical health	1 = Poor, 2 = Fair, 3 = Good
Satisfaction with the organizational position promotion mechanism	1 = Satisfied, 2 = Neutral, 3 = Dissatisfied
Satisfaction with the organizational professional title promotion mechanism	1 = Satisfied, 2 = Neutral, 3 = Dissatisfied
Average daily working hours	1 = <7 h, 2 = 7–9 h, 3 = > 9 h
Typical workload intensity	1 = Within capacity, 2 = Over capacity
Workload intensity during COVID-19	1 = Within capacity, 2 = Over capacity
Whether the supervisor is strict or not	1 = Yes, 2 = No
Get along well with colleagues and superiors or not	1 = Yes, 2 = Neutral or not
Conflict between personal values and work values	1 = No conflict, 2 = Neutral, 3 = Conflict
Effort-reward imbalance	1 = No, 2 = Yes
Family support for work	1 = Supportive, 2 = Average or non-supportive
Whether the work-life balance be achieved or not	1 = Yes, 2 = No
Stress level (score)	1 = ≤60, 2 = 61–80, 3 = > 80
Occurrence of job burnout	0 = No, 1 = Yes

**Table 5:** Binary logistic regression analysis on influencing factors of job burnout among the PHPs.

Variables	*β*	*S.E.*	*Waldχ* ^2^	*p*	*OR*	95% *CI*
Self-rated mental health	−0.831	0.327	6.464	0.011	0.436	0.230, 0.827
Workload intensity	1.645	0.554	8.834	0.003	5.183	1.751, 15.340
Family support for work	1.198	0.464	6.670	0.010	3.313	1.335, 8.222
Constant	0.342	1.273	0.072	0.788	1.407	—

CI, confidence interval; OR, odds ratio; PHPs, public health practitioners; S.E., standard error. “—” indicated that multivariable analysis did not provide numerical value.

### 3.5 Mediation effects of self-rated mental health and family support for work

The empirical studies have demonstrated that workload intensity exerts a direct influence on individuals’ mental health status (29), and the deterioration of mental health constitutes an important factor of burnout (30). In alignment with the Job Demands-Resources model (31), family support—as a critical dimension of social support—may moderate the relationship between workload and burnout. Given that self-rated mental health, family support for work, and workload intensity were identified as associated factors of burnout among PHPs, this study further investigated whether self-rated mental health and family support for work serve as mediating variables in the relationship between workload intensity and burnout. The mediation analyses were conducted using 5,000 Bootstrap resamples.

The results of mediation effects were summarized in Table 6. Increased workload indirectly elevated the likelihood of burnout by impairing self-rated mental health (indirect effect = 2.931, 95% *CI*: 1.111, 4.750). This suggested that higher workload exacerbated mental health deterioration, which in turn contributed to burnout. Additionally, elevated workload was associated with reduced family support for work (indirect effect = 1.609, 95% *CI*: −0.061, 3.280), further indirectly increasing the odds of burnout. However, self-rated mental health and family support for work did not reach statistical significance in mediating the effects between workload and job burnout among PHPs (*p* > 0.05).

**Table 6:** Mediation effects of self-rated mental health and family support for work.

Mediating pathways	Indirect effect	Bootstrap *S.E.*	*Z*	*p*	95% *CI*
Workload → self-rated mental health → burnout	2.931	0.928	3.16	0.002	1.111, 4.750
Workload → family support for work→ burnout	1.609	0.852	1.89	0.059	−0.061, 3.280
Workload → self-rated mental health → exhaustion	2.801	0.860	3.26	0.001	1.115, 4.486
Workload → self-rated mental health → cynicism	2.977	0.944	3.15	0.002	1.127, 4.826

CI, confidence interval; S.E., standard error.

To further examine the mediating roles of self-rated mental health and family support for work between workload intensity and the three dimensions of burnout, factors related to each dimension were analyzed (see Supplementary Material for details). The results revealed that self-rated mental health and typical workload intensity significantly influenced the exhaustion and cynicism dimensions, whereas family support for work was a key influencing factor for the inefficacy dimension. Consequently, the mediating effects of self-rated mental health on the relationships between workload intensity and both exhaustion and cynicism were tested. The findings, presented in Table 6, revealed that the indirect effects of workload intensity on both exhaustion dimension (indirect effect = 2.801, 95% *CI*: 1.115, 4.486) and cynicism dimension (indirect effect = 2.977, 95% *CI*: 1.127, 4.826) through diminished self-rated mental health were statistically significant (*p* < 0.05). Excessive workload indirectly elevated the odds of exhaustion and cynicism through its detrimental effects on mental health.

## 4 Discussion

This investigation revealed that more than half (50.90%) of the PHPs suffered from varying degrees of job burnout, predominantly characterized as mild burnout (prevalence of 27.48%), with moderate and severe burnout prevalence of 14.86 and 8.56%, respectively. Among the three dimensions of burnout, exhaustion emerged as particularly salient (prevalence of 32.88%). The factors influencing job burnout among the PHPs in Baoan District encompassed self-rated mental health status, workload intensity, and the family support for work. Individuals reporting poorer self-rated mental health, experiencing excessive workload, and receiving limited family support demonstrated a heightened risk of job burnout. And the mediation analysis revealed that neither self-rated mental health nor family support for work demonstrated statistically significant mediating effects in the relationship between workload and job burnout among PHPs.

Global empirical evidence underscored significant transregional commonality in burnout prevalence among PHPs. The prevalence of job burnout among rural physicians and community-based physicians engaged in public health initiatives were reported at 53.47 and 59.7%, respectively (23, 32). A cross-sectional survey conducted in 2018 involving public health service providers across six provinces in China indicated a job burnout prevalence of 58.06% (7), which was slightly elevated compared to the findings of this study. During the COVID-19 pandemic, a study in central China revealed a significant burnout rate of 62.6% among grassroots health workers (33). High burnout prevalence among healthcare professionals was also evident internationally, with 53.85% of personnel in Brazilian public hospitals experiencing burnout (34), and 66.2% of public health workers in the United States reporting similar conditions (35). In Tunisia, the job burnout prevalence among healthcare workers reached an alarming 77.9% (36). Max et al. conducted a survey among healthcare practitioners in the United Kingdom, Poland, and Singapore, finding an overall job burnout prevalence of 67% (37). A meta-analysis highlighted that the global prevalence of job burnout among the PHPs ranged from 10.5 to 85.2%, with a pooled prevalence of 42% during the COVID-19 pandemic and 35% in non-pandemic contexts (38). Like frontline clinical healthcare staff, the PHPs faced severe shortages and extremely difficult working conditions, which had led to their participation in multiple response and control activities, exacerbating the occurrence of job burnout. With the end of the COVID-19 pandemic, it was assumed that job burnout among the PHPs might have improved. However, due to the recent end of the COVID-19 pandemic, the current situation reflected in the survey might still be compounded by its aftermath.

Among the related factors of job burnout, we found that PHPs with poorer self-rated mental health had more job burnout than those with more optimal level. Notably, the association had been established between deteriorating self-rated mental health and increased job burnout risk. This aligned with a study conducted in Beijing, which identified mental health status as a significant risk factor for job burnout (39). However, that investigation employed standardized scales for assessing participants’ psychological conditions, whereas this study relied on self-assessment metrics. Additionally, findings from a survey of healthcare workers in Singapore revealed a noteworthy association between adverse psychological states, such as anxiety and depression, and job burnout (30). The COVID-19 pandemic had underscored the critical role of the PHPs, who were increasingly subjected to elevated pressure and workload demands. Despite the essential nature of their work, the PHPs often faced less attractive remuneration and career advancement opportunities compared to their clinical counterparts, coupled with low levels of societal recognition. This disparity can engender feelings of psychological dissonance, imbalance, and a sense of misalignment between effort and reward, ultimately contributing to heightened feelings of loss, anxiety, and depression. The excessive depletion of psychological resources, if not mitigated by timely external interventions, might lead to the manifestation of job burnout. The direct association between self-rated mental health and burnout remained robust underscoring the critical role of mental health in mitigating burnout. The pronounced indirect effects suggested that self-rated mental health mediated in the relationships between workload intensity and burnout. When disentangling burnout into its core dimensions, self-rated mental health also emerged as a critical mediator for exhaustion and cynicism. This suggested that workload-driven mental health deterioration predominantly manifested in emotional and attitudinal outcomes, underscoring the affective pathways of burnout. Therefore, it was imperative to acknowledge and address the mental health challenges faced by the PHPs to ensure their well-being and enhance the sustainability of public health initiatives.

Overwork was a significant risk factor for job burnout, a phenomenon that had been documented in studies examining job burnout among personnel from disease prevention and control in Jilin Province and medical staff from Liaoning Province (14, 40). Research conducted in the United States indicated that work overload increased the likelihood of job burnout among healthcare professionals by a factor of 2.90 (41), with the incidence of job burnout rising proportionally with the extension of working hours (42). Additionally, healthcare workers in Singapore and Bangladesh who endured long shifts of 8 hours or more exhibited elevated levels of job burnout (30, 43), further corroborating the effect of excessive workload on job burnout occurrence. Indeed, as early as 1997, work overload was recognized as a fundamental cause of job burnout (14). The high-pressure, high-demand work environment imposed an excessive strain on the mental and physical resources of the PHPs, resulting in heightened job burnout levels. Remarkably, in this study, PHPs with work overload exhibited 5.183 times higher odds of occupational burnout compared to those without overload (95% *CI*, 1.751, 15.340). Specifically, 88.1% of overloaded PHPs developed burnout, indicating an elevated event rate (e.g., high burnout prevalence) that inherently increase variability in parameter estimates. Simultaneously, the limited sample size amplified estimation uncertainty, further contributing to broader confidence interval.

The mediation analysis indicated that elevated workload indirectly increased the likelihood of exhaustion and cynicism by exacerbating mental health deterioration. This aligns with the Job Demands-Resources model, wherein chronic work demands deplete psychological resources, heightening vulnerability to emotional exhaustion and negative attitudes toward work roles (31). And increased workload might indirectly elevate burnout risk through a plausible pathway: i.e., reduced family support for work. While these indirect effects aligned with theoretical expectations—suggesting that workload amplified burnout by eroding family support—they narrowly missed conventional statistical significance thresholds. This marginal significance implied that larger sample sizes or longitudinal designs might be required to confirm these mediation pathways. The mediating effects elucidated plausible mechanism by which excessive workload contributed to occupational burnout. And the mechanisms linking high workload to burnout in PHPs were inherently complex. Firstly, physiological fatigue accumulation—stemming from prolonged exposure to high-intensity tasks—might manifest as sleep deprivation, which directly impaired emotional regulation. Secondly, sustained high workload predisposed individuals to cognitive overload and decision fatigue, depleting mental resources required for effective task execution. Thirdly, excessive workload often correlated with an imbalance between work and personal life, exacerbating psychological burdens by limiting opportunities for recovery and personal fulfillment. Notably, in the current occupational context, the burdensome nature of such work typically did not translate into increased compensation or external support, thereby adversely affecting overall job satisfaction and experience.

The extent of family support significantly influenced the prevalence of job burnout among the PHPs. Wang et al. had elucidated that perceived social support, particularly from familial sources, served as a moderating factor between EE and subjective well-being, thereby effectively diminishing the risk of job burnout (44). A study conducted in the central region of China highlighted that work-family support acted as a protective factor against job burnout in primary healthcare workers (33). Previous research had consistently demonstrated the inhibitory effects of family and social support on job burnout (45). When the PHPs were laden with high-intensity household responsibilities after work or when their professional challenges were not understood or supported by family members, their levels of psychological relaxation were significantly reduced, which could adversely affect their work engagement. In contrast, the PHPs who enjoyed robust family support were better equipped to leverage this support to swiftly navigate challenges encountered in their professional roles. Family support provided employees with social and emotional resources, thereby reducing their susceptibility to burnout, particularly exhaustion, in the workplace. This assertion was corroborated by research conducted during the COVID-19 pandemic in South Korea, which found that family support can positively influence job performance by alleviating emotional fatigue (18). This phenomenon could be interpreted through the lens of the work- home resources model, which posited that familial resources could spill over into the occupational domain (46). Undeniably, adequate support from family members could mitigate the work-related pressures faced by the PHPs, reduce negative experiences within the workplace, and ultimately lessen the severity of job burnout. In this study, family support for work was uniquely associated with burnout and the inefficacy dimension. Despite theoretical expectations, it failed to mediate the workload-burnout relationship. It is reasonable to hypothesize that the protective role of family support may mitigate perceptions of inefficacy, however, its capacity to counteract workload-induced strain might be less pronounced than theoretically anticipated. This tentative hypothesis necessitates rigorous empirical validation through future investigation.

Job burnout results from a complex interplay of factors, necessitating integrated interventions that address physiological, psychological, and family support dimensions. Empirical evidence underscores the need for systemic strategies to mitigate this multifactorial challenge among the PHPs in the post-COVID-19 era. Firstly, provide psychosocial support to PHPs. The phenomenon of job burnout is characterized by dynamic fluctuations and is closely intertwined with mental health. Consequently, it is imperative to conduct annual assessments of the combined status of job burnout and mental health among the PHPs. Such evaluations can elucidate the progression of job burnout and facilitate the implementation of targeted interventions. And it is indispensable to strengthen workplace resources, such as counseling services and peer support networks, to buffer against mental health decline. Secondly, manage workload effectively. The organizational and management entities should prioritize the provision of essential resources, support systems, and training opportunities for employees. Implementing flexible work arrangements, effectively managing workload, and promoting initiatives that encourage work-life balance are critical strategies that can mitigate the risks associated with work overload and reduce its detrimental effects on employee burnout. Thirdly, develop family engagement programs. The PHPs should be encouraged to strengthen communication with their family members, particularly during periods of increased workload. Effective communication is able to foster greater understanding and support from families, allowing these professionals to allocate more energy to their work responsibilities. Furthermore, organizations should cultivate a work environment that is supportive of family needs, with health administrative bodies and public health institutions considering the introduction of supportive policies and flexible work arrangements for staff experiencing family-related work disruptions. Such measures are essential for enhancment of the overall well-being and effectiveness of the PHPs.

The research presented both limitations and strengths. Regarding the limitations, firstly, the limited number of personnel engaged in public health services in this region resulted in a small sample size. Secondly, because participation was based on voluntary enrollment, the sample might exhibit selection bias. Thirdly, some variables in the questionnaire, such as mental health status, were not measured using standardized scales, which might affect the reliability of the findings. Lastly, as a cross-sectional study, this research was constrained in its ability to establish causal relationships and temporal sequences. With respect to the strengths, notably, this study was one of the few investigations exploring the status and influencing factors of job burnout among the PHPs who had experienced the COVID-19 pandemic. It offers valuable insights into the psychological well-being and work engagement of the PHPs following significant public health events, thereby contributing to the understanding of their challenges and informing future support strategies.

## 5 Conclusion

This study highlighted that job burnout was a prevalent issue among the PHPs after the COVID-19 pandemic. A total of 50.90% of participants reported experiencing job burnout. The job burnout of the PHPs was affected by self-rated mental health, workload intensity, and the family support for works. To effectively mitigate the incidence of job burnout in this population, it was imperative for health management authorities, public health institutions, and individuals to collaboratively develop and implement multifaceted strategies and interventions. These efforts should focued on reducing associated risk factors to ultimately enhance the well-being and resilience of employees within the public health sector.

## Data Availability

The raw data supporting the conclusions of this article will be made available by the authors, without undue reservation.
