# Supplementary material for: Job burnout among public health practitioners in urban China: insights from the post-COVID-19 pandemic context
Source: Front Public Health. 2025 May 21;13:1518114. doi: 10.3389/fpubh.2025.1518114 (PMC12133727; doi:10.3389/fpubh.2025.1518114)
Supplement: Supplementary file 1 [file Table_1.docx]

**Supplementary Table 1 Binary logistic regression analysis on influencing factors of exhaustion among the PHPs**

| Variables | *β* | *S.E.* | *Waldχ*^2^ | *P* | *OR* | 95% *CI* |
| --- | --- | --- | --- | --- | --- | --- |
| Marital status | 3.013 | 0.915 | 10.838 | 0.001 | 20.342 | 3.384, 122.27 |
| Number of children | 1.101 | 0.480 | 5.263 | 0.022 | 3.007 | 1.174, 7.704 |
| Self-rated mental health | -0.773 | 0.338 | 5.245 | 0.022 | 0.461 | 0.238, 0.894 |
| Typical workload intensity | 2.510 | 0.589 | 18.148 | 0.000 | 12.307 | 3.878, 39.058 |
| Conflict between personal values and work values | 0.958 | 0.386 | 6.142 | 0.013 | 2.606 | 1.222, 5.557 |
| Whether the work-life balance be achieved or not | 2.602 | 1.062 | 5.999 | 0.014 | 13.492 | 1.682, 108.25 |
| Stress level | 1.184 | 0.294 | 16.177 | 0.000 | 3.268 | 1.835, 5.82 |
| Constant | -13.459 | 2.889 | 21.705 | 0.000 | 0.000 | — |

Note: Abbreviations: CI, confidence interval; OR, odds ratio; PHPs, public health practitioners; S.E., standard error.

“—” indicated that multivariable analysis did not provide numerical value.

**Supplementary Table 2 Binary logistic regression analysis on influencing factors of cynicism among the PHPs**

| Variables | *β* | *S.E.* | *Waldχ*^2^ | *P* | *OR* | 95% *CI* |
| --- | --- | --- | --- | --- | --- | --- |
| Position titles | -1.221 | 0.569 | 4.596 | 0.032 | 0.295 | 0.097, 0.901 |
| Self-rated mental health | -1.355 | 0.399 | 11.513 | 0.001 | 0.258 | 0.118, 0.564 |
| Satisfaction with the organizational position promotion mechanism | 0.946 | 0.384 | 6.056 | 0.014 | 2.575 | 1.212, 5.468 |
| Typical workload intensity | 1.963 | 0.463 | 17.979 | 0.000 | 7.118 | 2.873, 17.635 |
| Conflict between personal values and work values | 0.957 | 0.415 | 5.331 | 0.021 | 2.605 | 1.156, 5.871 |
| Constant | -1.780 | 1.782 | 0.997 | 0.318 | 0.169 | — |

Note: Abbreviations: CI, confidence interval; OR, odds ratio; PHPs, public health practitioners; S.E., standard error.

“—” indicated that multivariable analysis did not provide numerical value.

**Supplementary Table 3 Binary logistic regression analysis on influencing factors of inefficacy among the PHPs**

| Variables | *β* | *S.E.* | *Waldχ*^2^ | *P* | *OR* | 95% *CI* |
| --- | --- | --- | --- | --- | --- | --- |
| Conflict between personal values and work values | 0.599 | 0.275 | 4.760 | 0.029 | 1.821 | 1.063, 3.120 |
| Family support for work | 0.980 | 0.363 | 7.296 | 0.007 | 2.665 | 1.309, 5.426 |
| Constant | -3.072 | 0.538 | 32.551 | 0.000 | 0.169 | — |

Note: Abbreviations: CI, confidence interval; OR, odds ratio; PHPs, public health practitioners; S.E., standard error.

“—” indicated that multivariable analysis did not provide numerical value.
